# Unliganded TRs regulate growth and developmental timing during early embryogenesis: evidence for a dual function mechanism of TR action

**DOI:** 10.1186/2045-3701-5-8

**Published:** 2015-03-02

**Authors:** Paul M Yen

**Affiliations:** Laboratory of Hormonal Regulation, Cardiovascular and Metabolic Disorders Program, 8 College Road, Level 8D14, Singapore, 169857 Singapore

**Keywords:** Thyroid hormone receptor, Vertebrate postembryonic development, Developmental timing, Organogenesis

## Abstract

Recent genetic studies in the anuran Xenopus tropicalis reveal surprising new roles of thyroid hormone receptor in regulating growth and developmental timing in the absence of thyroid hormone.

## Text

Thyroid hormone (T_3_) regulates adult metabolism and postembryonic development in vertebrates [1]. T_3_ functions mainly by binding to its receptors (TRs) to regulate gene expression. There are two TR genes, TRα and TRβ, with TRα expressed more ubiquitously and earlier during development than TRβ. TRs regulate target gene transcription in a T_3_-dependent manner. For T_3_-inducible genes that are positively regulated, TRs recruit coactivator and corepressor complexes in the presence and absence of T_3_, respectively. This leads to chromatin remodeling and changes in histone modifications to activate and repress target gene transcription [1–3]. This dual function mechanism suggests that both liganded and unliganded TRs have distinct physiological roles, particularly during embryonic stages when the fetus is exposed to little or no T_3_.

During mammalian development, TRα mRNA is detected earlier than endogenous T_3_, suggesting that TRs are mainly unliganded during these early stages. However, it has been challenging to ascertain whether unliganded TRs have transcriptional or physiological functions in a convincing manner since the transplacental supply of maternal T_3_ to the fetus complicates any analysis in mammalian systems. In addition, it is difficult to determine the functional role of unliganded TRs since it is not easy to manipulate their actions pharmacologically or genetically due to the uterine enclosure of mammalian embryos.

In two recent articles published in *Endocrinology*, the laboratories of Yun-Bo Shi of *Eunice Kennedy Shriver* National Institute Child Health and Human Development, and Daniel R. Buchholz of University of Cincinnati used *Xenopus tropicalis* as a model system to overcome some of these problems in studying the action of unliganded TRs during embryogenesis. Their findings have revealed novel functions for unliganded TRα in regulating embryonic animal development by taking advantage of the fact that anurans undergo biphasic development before and after metamorphosis. Accordingly, annurans first undergo embryogenesis to produce free-living tadpoles. After a period of growth, the tadpoles metamorphose into frogs via processes that are regulated by T_3_[4]. Similar to mammals, TRα mRNA is expressed very early and reaches high levels of expression by the end of embryogenesis when the feeding tadpole is formed and before synthesis of TH by the mature tadpole. Based on these and other observations, Shi’s group proposed a dual function model for TR during frog development over a decade ago [5]. Initially, T_3_-inducible genes are expressed at basal levels during early embryogenesis when there is little T_3_ and TR expression. At the end of embryogenesis, these genes are no longer needed for development; thus, a correspondingly high level of TR expression occurs during this stage in the relative absence of T_3_ that, in turn, leads to transcriptional repression of target genes. Later, as T_3_ becomes available, liganded TRs then activate the expression of T_3_-inducible genes that will play key roles in metamorphosis. Although extensive studies have demonstrated a critical role for liganded TRs in anuran development [6], there has been little direct evidence supporting the involvement of unliganded TRs in the transcriptional regulation of target genes *in vivo*.

To address this issue, the Shi and Buchholz groups ingeniously made use of the recently developed TALEN (transcriptional activator-like effector nuclease) method to investigate the role of TRα during the development of the diploid anuran Xenopus tropicalis. Both groups independently designed TALENs to specifically to knock down the TRα gene. Using complementary approaches, both teams demonstrated that removing TRα affected developmental timing in premetamorphic tadpoles, causing the animals to initiate metamorphosis earlier than their wild type siblings [7, 8]. Consistent with the dual function model for TR, T_3_-target genes were de-repressed during the pre-metamorphic stages in TRα knockdown embryos since gene expression was higher than those in wild type embryos that expressed TRα during these same stages. Additionally, the knockdown embryos were resistant to exogenous T_3_ treatment and underwent delayed metamorphoses. Surprisingly, Shi’s group made the unexpected discovery that knockdown of TRα enhanced tadpole growth in premetamorphic tadpoles. This growth most likely occurred due to de-repression (*i.e.*, increase) of growth hormone gene expression. These two studies independently demonstrated the validity of the dual function mechanism of TRα during anuran development as they not only showed the critical role of endogenous TRα in mediating the metamorphic effect of T_3_ but also revealed novel functions of unliganded TRα, that control both tadpole growth rate and timing of metamorphosis during post-embryonic development.

Vertebrate development is evolutionally conserved with frog metamorphosis resembling the postembryonic development period in mammals as the latter occurs shortly after birth and is accompanied by a burst of high production and circulating levels of T_3_[4]. Likewise, during early embryogenesis the fetus is in a relatively “hypothyroid” state as the fetal thyroid gland has not been formed yet and placental deiodinase III protects the fetus from exposure to maternal T_3_. It is likely that unliganded TRα plays major roles in repressing the transcription of important target genes during this period before they are activated later during pregnancy and the neonatal period. Therefore, the novel findings from these two studies strongly suggest that unliganded TRs may play similar roles in the regulation of embryo growth and the timing of organ development and maturation in mammals during the stages when TR is expressed but there is little or no fetal exposure to T_3_. They also provide new insight into some of the developmental consequences within the fetus during iodine deficiency during pregnancy, congenital hypothyroidism, and maternal thyroid dysfunction.
